# Cardiometabolic Disease Education and Health Services Among Public High Schools

**DOI:** 10.1001/jamanetworkopen.2026.36060

**Published:** 2026-09-25

**Authors:** Ibrahim Zaganjor, Ryan Saelee, Ahlia Sekkarie, Meda E. Pavkov, Alain K. Koyama, Jeong Ha Choi, Stephen Onufrak, Daesung Choi, Sola Han, Kathleen H. Krause

**Affiliations:** 1 Division of Diabetes Translation, National Center for Chronic Disease Prevention and Health Promotion, Centers for Disease Control and Prevention, Atlanta, Georgia; 2 Division of Adolescent and School Health, National Center for Chronic Disease Prevention and Health Promotion, Centers for Disease Control and Prevention, Atlanta, Georgia; 3 Division for Heart Disease and Stroke Prevention, National Center for Chronic Disease Prevention and Health Promotion, Centers for Disease Control and Prevention, Atlanta, Georgia; 4 Division of Nutrition, Physical Activity, and Obesity, National Center for Chronic Disease Prevention and Health Promotion, Centers for Disease Control and Prevention, Atlanta, Georgia

## Abstract

**Importance:**

There is a paucity of research on school-based health education and services for cardiometabolic conditions, particularly at the national level.

**Objective:**

To describe the health education and health services related to cardiometabolic health provided by US public high schools.

**Design, Setting, and Participants:**

This survey study included US public high schools who responded to the principal and lead health education teacher surveys as part of the School Health Profiles data collection in 2022. Data were analyzed between September 2025 and July 2026.

**Exposures:**

School-level characteristics included enrollment size, urbanicity, region, neighborhood income-to-poverty ratio, and racial and ethnic composition.

**Main Outcomes and Measures:**

Primary outcomes were health education provided to students (and separately) to parents and families on chronic disease prevention, nutrition, and physical activity and provision of school-based health services. These services included identifying and tracking students with a current diagnosis of diabetes, hypertension, or obesity; providing referrals for those confirmed or suspected to have these cardiometabolic conditions; and providing a full- or a part-time registered nurse, a school-based health center, daily medication administration for students with chronic conditions, case management for students with chronic conditions, and an insurance protocol for students with chronic conditions.

**Results:**

A total of 3371 high schools were included in the principal survey (776 small, 1422 medium, and 1173 large) and 3044 high schools were included in the teacher survey (641 small, 1321 medium, and 1082 large). In 2022, 89.0% (95% CI, 87.4%-90.3%), 95.5% (95% CI, 94.3%-96.4%), and 98.1% (95% CI, 97.3%-98.7%) of schools provided health education on chronic disease prevention, nutrition, and physical activity, respectively, to students, while 43.5% (95% CI, 41.3%-45.7%), 50.9% (95% CI, 48.7%-53.1%), and 50.8% (95% CI, 48.6%-53.0%) provided information on chronic disease prevention, nutrition, and physical activity, respectively, to families. Schools tracked diabetes (96.3%; 95% CI, 95.5%-97.0%) more frequently than hypertension (64.9%; 95% CI, 63.0%-66.6%) and obesity (38.6%; 95% CI, 36.8%-40.4%); similarly, schools provided referrals for diabetes (52.1%; 95% CI, 50.3%-54.0%) more frequently than for hypertension (48.5%; 95% CI, 46.6%-50.4%) and obesity (41.6%; 95% CI, 39.8%-43.5%). Most schools reported having a full- or part-time registered nurse (90.9%; 95% CI, 89.8%-91.9%), but only 29.3% (95% CI, 27.7%-31.1%) reported having a school-based health center.

**Conclusions and Relevance:**

In this survey study of US public high schools, gaps in cardiometabolic health education and services were identified. These findings may inform future programs to improve cardiometabolic health among adolescents.

## Key Points

**Question:**

What is the availability of school-based education and services related to cardiometabolic health among US public high schools, and does this vary by school-level sociodemographic characteristics?

**Findings:**

In this survey study, 3371 US public high schools responded to a principal survey and 3044 high schools responded to a teacher survey; nutrition, physical activity, and chronic disease prevention education was prevalent for high school students but less available for parents and family members. The availability of health services varied by several school-level characteristics, notably region and racial and ethnic composition.

**Meaning:**

These findings highlight the gaps in school-based resources that could reduce the burden and impact of cardiometabolic conditions among US adolescents.

## Teaser

This survey study describes the provision of cardiometabolic health–related education and services by US public high schools to students and families.

## Introduction

Cardiometabolic conditions are becoming increasingly common among children and adolescents in the US.^1,2^ Findings from the National Health and Nutrition Examination Survey^3^ and SEARCH for Diabetes in Youth study have documented increases in obesity and type 2 diabetes among children and adolescents in recent decades.^4^ These trends coincide with a decline in healthy behaviors, such as daily fruit and vegetable consumption, adequate physical activity, and sleep.^5^

Select school-based programs and interventions, when successfully implemented, have demonstrated improvements in physical activity and nutritional quality of food and meals available at schools.^6,7,8^ Because children and adolescents spend a substantial amount of time at school, school-based health services and health education could help improve cardiometabolic health outcomes among school-aged youths. School-based programs are particularly unique because they can help target behaviors and cardiometabolic risk factors (eg, nutrition and physical activity) within stable and existing structures and provide an avenue to reach students and their parents.^9^ Additionally, they may help children and adolescents improve educational outcomes, such as absenteeism.^10^

A critical gap in the literature is an inventory of school-based education and services currently being offered to children and adolescents to complement the extant research on youth health behaviors and conditions. An analysis of school programs implemented to facilitate prevention and management of cardiometabolic conditions among students is timely and needed to meet national efforts for improving the health and well-being of youths. Thus, this study aimed to describe the health education and school-based health services related to cardiometabolic conditions provided by US public high schools.

## Methods

### Data Source

This survey study was not subject to institutional review board approval due to being a secondary analysis in accordance with federal law (45 CFR 46). Reporting follows the American Association for Public Opinion Research (AAPOR) reporting guideline.

Data related to health education and services came from the School Health Profiles (hereafter referred to as *Profiles*) surveillance system developed by the Centers for Disease Control and Prevention (CDC) to assess school health policies and practices among secondary schools across various jurisdictions, including states, school districts, US territories, and tribes.^11^ Two surveys were administered per school: one to the school principal and the other to the lead health education teacher. In 2022, schools from 45 states and Washington DC participated; these data were combined and reweighted to produce nationally representative estimates of schools with students in grades 6 to 12. Additional details on survey sampling, administration, response rates, and weighting for the principal and lead health education teacher surveys are described elsewhere.^12^

Information on demographic characteristics of schools in Profiles was obtained from the 2021-2022 National Center for Education Statistics (NCES) Common Core of Data (CCD), which is the Department of Education’s primary data source about public elementary and secondary schools in the US, and the NCES Education Demographic and Geographic Estimates (EDGE) program. Profiles data were linked to CCD and EDGE via a unique school identifier. This Profiles dataset with unique identifiers is protected by an assurance of confidentiality and is only accessible to staff within the Division of Adolescent and School Health at the CDC.

Because the primary purpose of this analysis was the school environment of high school aged students, only schools with students in grades 9 to 12 were included. Schools that could not be matched due to missing or invalid identifiers, lacked enrollment data, or had specialized curricula (eg, schools that primarily focus on vocational or technical education) were excluded. In total, 51 of 3422 high schools (1.5%) and 38 of 3082 (1.2%) high schools were excluded from the final analytic sample for the principal and teacher surveys, respectively.

### Study Measures

Questions on health education and services related to the prevention and treatment of cardiometabolic conditions were selected from the surveys for this analysis. From the principal survey, the analyses included the questions with response options (yes or no) to whether the school routinely uses school records to identify and track students with a current diagnosis of diabetes (unspecified type), hypertension, or obesity, and whether the school provides referrals for students with confirmed or suspected diabetes (unspecified type), hypertension, or obesity. In addition, the analyses incorporated questions about whether the school had a full-time or a part-time registered nurse, a school-based health center (ie, school-based health centers on school campus where enrolled students can receive primary care, including diagnostic and treatment services), daily medication administration for students with chronic conditions, case management for students with chronic conditions, and an insurance protocol for students with chronic conditions. From the lead health education teacher survey, the analyses included questions asking whether teachers have tried to increase student knowledge on chronic disease prevention, nutrition, and physical activity and whether teachers have provided parents and families with health information designed to increase parent and family knowledge on chronic disease prevention, nutrition, and physical activity. Demographic characteristics included school enrollment size (small, <300 students; medium, 300-999 students; large, ≥1000 students); urbanicity (city, suburban, town, and rural)^13^; region (Northeast, Midwest, South, and West)^14^; neighborhood income-to-poverty ratio (IPR; <200%, 200% to <300%, and ≥300%); and racial and ethnic composition, with a majority being defined as more than 50% of the school (Black majority, Hispanic majority, White majority, other majority, and no racial and ethnic majority). Race and ethnicity data were ascertained from the Public Elementary and Secondary School Universe Survey component of the NCES-CCD, which collects student enrollment for every grade in the school and the number of students in each of the following 7 racial and ethnic categories: American Indian or Alaska Native, Asian, Black or African American, Hispanic or Latino, Native Hawaiian or Other Pacific Islander, 2 or more races, and White.^15^ In the present study, the other majority category includes schools with American Indian or Alaska Native, Asian, Native Hawaiian or Other Pacific Islander, 2 or more races, and unspecified race majorities; race categories were defined to be mutually exclusive of Hispanic ethnicity.

### Statistical Analysis

Weighted percentages with 95% CIs were estimated for affirmative responses to the questions for the overall school population and by demographic characteristics. Prevalence estimates for chronic disease health education and services among other majority schools were suppressed due to the small sample size, resulting in unreliable estimates. Prevalence estimates (or their complements [ie, 1 – percentage]) with a relative standard error greater than 30%, suggesting potential statistical unreliability, were flagged.

In a supplemental analysis, logistic regression models with predictive marginals were used to estimate unadjusted prevalence ratios (PRs) and adjusted PRs (aPRs) with 95% CIs to compare the prevalence of affirmative responses to each question by demographic subgroup. Adjusted models controlled for school enrollment, urbanicity, region, neighborhood IPR, and racial and ethnic composition. Differences were significant (α level of .05) if the 95% CI did not include the null value. Because these comparisons were completed for hypothesis-generating purposes, no corrections for multiple comparisons were conducted.

Data were analyzed between September 2025 and July 2026. All analyses accounted for the complex survey design and weighting of Profiles and were conducted using SAS-callable SUDAAN version 11.0.4 (Research Triangle Institute).

## Results

A total of 3371 high schools were included for analysis in the principal survey sample (776 small, 1422 medium, and 1173 large), and 3044 high schools were included for analysis in the teacher survey sample (641 small, 1321 medium, and 1082 large) (Table 1). In the principal survey, schools were most frequently medium sized, located in a rural area, in neighborhoods with an IPR of 300% or greater, and in the South of the US. White student majority schools were most common (59.8%; 95% CI, 58.0%-61.5%), followed by Hispanic majority (15.7%; 95% CI, 14.2%-17.3%), and schools with no racial and ethnic majority (15.6%; 95% CI, 14.3%-17.0%). Black majority and other majority schools represented 7.2% (95% CI, 6.3%-8.2%) and 1.7% (95% CI, 1.3%-2.2%) of the principal survey sample, respectively. The distribution of school sociodemographic characteristics was similar for the teacher survey sample.

**Table 1.** Characteristics of Public High Schools (Grades 9-12): School Health Profiles Principal and Lead Health Education Teacher Surveys, US, 2022

Characteristic^a^	Principal survey		Lead health education teacher survey	
	No.	Weighted % (95% CI)	No.	Weighted % (95% CI)
Overall, nationwide	3371	NA	3044	NA
School enrollment (grades 9-12)^b^				
Small	776	19.6 (18.4-20.9)	641	19.3 (17.8-20.9)
Medium	1422	40.8 (39.1-42.6)	1321	40.5 (38.5-42.5)
Large	1173	39.5 (37.7-41.3)	1082	40.3 (38.2-42.4)
Urbanicity				
City	588	21.1 (19.5-22.7)	539	21.1 (19.4-22.9)
Suburban	857	27.5 (25.9-29.2)	785	27.4 (25.6-29.4)
Town	632	16.8 (15.6-18.1)	572	16.6 (15.2-18.0)
Rural	1294	34.6 (33.0-36.3)	1148	34.9 (33.0-36.9)
Region^c^				
Northeast	682	18.6 (17.5-19.7)	602	18.8 (17.5-20.2)
Midwest	946	27.1 (25.9-28.3)	917	26.9 (25.6-28.2)
South	1112	35.5 (34.0-36.9)	932	35.1 (33.4-36.8)
West	631	18.8 (17.5-20.1)	593	19.3 (17.8-20.8)
Neighborhood income-to-poverty ratio				
<200%	513	16.5 (15.2-17.9)	442	16.3 (14.8-18.0)
200% to <300%	1347	39.0 (37.2-40.7)	1216	39.1 (37.0-41.1)
≥300%	1511	44.5 (42.7-46.3)	1386	44.6 (42.5-46.7)
Racial and ethnic composition^d^				
Black majority	235	7.2 (6.3-8.2)	223	7.0 (6.0-8.2)
Hispanic majority	338	15.7 (14.2-17.3)	296	16.0 (14.3-17.8)
White majority	2254	59.8 (58.0-61.5)	2066	59.6 (57.5-61.6)
No majority	472	15.6 (14.3-17.0)	402	15.6 (14.1-17.3)
Other majority^e^	72	1.7 (1.3-2.2)	57	1.8 (1.3-2.6)

Abbreviations: NA, not applicable.

^a^ School characteristics data were ascertained from the US Department of Education National Center for Education Statistics (NCES) 2021-2022 Common Core of Data^15^and the NCES Education Demographic and Geographic Estimates Program.^13^

^b^ Small indicates less than 300 students; medium, 300 to 999 students; large, 1000 students or more.

^c^ Defined by the US Census Bureau.^14^

^d^ Race categories exclude persons of Hispanic ethnicity. Majority represents more than 50% of enrollment across grades 9 to 12.

^e^ Other majority category includes schools with American Indian or Alaska Native, Asian, Native Hawaiian or Other Pacific Islander, 2 or more races, and unspecified race majorities.

### School-Based Tracking and Referrals of Cardiometabolic Conditions

Overall, 96.3% (95% CI, 95.5%-97.0%), 64.9% (95% CI, 63.0%-66.6%), and 38.6% (95% CI, 36.8%-40.4%) of schools used school records to identify and track students with a current diagnosis of diabetes, hypertension, and obesity, respectively (Table 2). In contrast, referrals for students confirmed or suspected to have these conditions were provided in only 52.1% (95% CI, 50.3%-54.0%) of schools for diabetes, 48.5% (95% CI, 46.6%-50.4%) of schools for hypertension, and 41.6% (95% CI, 39.8%-43.5%) of schools for obesity overall. The prevalence of providing referrals for each of these conditions was distinctively lower among rural schools (Table 2). White majority schools identified and tracked hypertension and obesity less frequently than Black and Hispanic majority schools and had a notably lower prevalence of providing referrals for these conditions. Schools in the Northeast had the highest prevalence of identifying, tracking, and providing referrals for all 3 cardiometabolic conditions. Unadjusted PRs and aPRs estimating differences by each school-level characteristic are reported in eTable 1 in the Supplement.

**Table 2.** Percentage of Public High Schools (Grades 9-12) Tracking and Providing Referrals for Cardiometabolic Conditions Overall and by Select School Characteristics: School Health Profiles Principal Survey, 2022

Characteristic^a^	Use school records to identify and track students with current diagnosis, weighted % (95% CI)^b^			Provide referrals for confirmed or suspected chronic conditions, weighted % (95% CI)^c^		
	Diabetes	Hypertension	Obesity	Diabetes	Hypertension	Obesity
Overall, nationwide	96.3 (95.5-97.0)	64.9 (63.0-66.6)	38.6 (36.8-40.4)	52.1 (50.3-54.0)	48.5 (46.6-50.4)	41.6 (39.8-43.5)
School enrollment^d^						
Small	95.3 (93.4-96.7)	59.6 (55.9-63.1)	34.8 (31.4-38.4)	50.1 (46.4-53.8)	46.4 (42.8-50.1)	39.9 (36.4-43.5)
Medium	96.5 (95.4-97.4)	68.4 (65.7-70.9)	41.1 (38.4-43.9)	53.1 (50.3-55.9)	48.4 (45.6-51.3)	42.3 (39.5-45.2)
Large	96.5 (94.9-97.6)	63.9 (60.6-67.1)	37.8 (34.7-41.0)	52.2 (48.9-55.5)	49.7 (46.4-53.0)	41.8 (38.6-45.0)
Urbanicity						
City	96.0 (94.1-97.3)	66.0 (61.5-70.2)	44.5 (40.0-49.0)	62.0 (57.5-66.2)	57.7 (53.2-62.1)	53.5 (49.0-57.9)
Suburban	96.0 (93.7-97.5)	66.5 (62.9-70.0)	41.9 (38.3-45.6)	50.5 (46.7-54.4)	47.9 (44.1-51.7)	42.0 (38.4-45.8)
Town	97.7 (96.5-98.5)	66.5 (62.6-70.2)	33.8 (29.9-37.9)	54.2 (50.0-58.3)	50.3 (46.1-54.5)	41.9 (37.8-46.1)
Rural	96.0 (94.7-97.0)	62.0 (59.0-64.9)	34.6 (31.8-37.4)	46.4 (43.4-49.4)	42.5 (39.6-45.5)	33.9 (31.2-36.7)
Region^e^						
Northeast	98.1 (96.6-98.9)	78.0 (74.6-81.2)	63.2 (59.2-66.9)	63.8 (59.7-67.7)	62.2 (58.1-66.1)	57.5 (53.4-61.5)
Midwest	96.8 (95.6-97.6)	60.7 (57.7-63.7)	30.4 (27.7-33.4)	50.4 (47.3-53.5)	44.7 (41.7-47.8)	37.4 (34.4-40.5)
South	95.7 (94.1-97.0)	65.8 (62.5-68.9)	36.4 (33.3-39.6)	46.8 (43.5-50.1)	43.9 (40.6-47.2)	36.5 (33.4-39.7)
West	95.0 (92.3-96.8)	56.7 (51.8-61.5)	31.1 (26.7-35.9)	53.7 (48.8-58.6)	49.8 (44.9-54.8)	42.3 (37.5-47.3)
Neighborhood income-to-poverty ratio						
<200%	95.5 (93.1-97.1)	67.9 (63.2-72.3)	41.1 (36.5-45.9)	58.6 (53.7-63.3)	55.2 (50.4-60.0)	48.0 (43.2-52.8)
200% to <300%	96.4 (95.0-97.4)	66.8 (64.0-69.5)	39.1 (36.2-42.0)	50.3 (47.4-53.3)	47.5 (44.6-50.5)	40.4 (37.5-43.4)
≥300%	96.5 (95.3-97.5)	62.0 (59.2-64.7)	37.1 (34.5-39.9)	51.3 (48.4-54.1)	46.9 (44.0-49.7)	40.3 (37.6-43.1)
Racial and ethnic composition^f^						
Black majority	92.2 (87.4-95.3)	73.4 (66.5-79.3)	52.3 (45.2-59.3)	54.8 (47.5-61.8)	53.6 (46.4-60.7)	47.0 (40.0-54.1)
Hispanic majority	95.7 (92.2-97.6)^g^	68.8 (63.0-74.1)	42.7 (37.1-48.6)	60.6 (54.6-66.3)	57.1 (51.1-62.8)	54.0 (48.1-59.8)
White majority	96.9 (96.0-97.5)	63.0 (60.8-65.1)	35.5 (33.4-37.6)	48.2 (46.0-50.4)	44.2 (42.0-46.4)	36.5 (34.4-38.6)
No majority	96.4 (93.9-97.9)	64.4 (59.3-69.1)	40.6 (35.7-45.7)	56.5 (51.3-61.5)	53.4 (48.3-58.5)	46.8 (41.7-51.9)

^a^ School characteristics data were ascertained from the US Department of Education, National Center for Education Statistics (NCES), 2021-2022 Common Core of Data^15^ and the NCES Education Demographic and Geographic Estimates Program.^13^

^b^ Based on the survey question, “Does your school routinely use school records to identify and track students with a current diagnosis of the following chronic conditions? School records might include student emergency cards, medication records, health room visit information, emergency care and daily management plans, physical exam forms, or parent notes.”

^c^ Based on the survey question, “Does your school provide referrals to any organizations or health care professionals not on school property for students diagnosed with or suspected to have any of the following chronic conditions? Include referrals to school-based health centers, even if they are located on school property.”

^d^ Small indicates less than 300 students; medium, 300 to 999 students; large, 1000 students or more.

^e^ Defined by the US Census Bureau.^14^

^f^ Race categories exclude persons of Hispanic ethnicity. Majority represents more than 50% of enrollment across grades 9 to 12. The other majority category (includes schools with American Indian or Alaska Native, Asian, Native Hawaiian or Other Pacific Islander, 2 or more races, and unspecified race majorities) was suppressed due to sample size.

^g^ Indicates the complement of the estimate (1 – percentage) may be unreliable (relative standard error >30%).

### School Health Services

Regarding specific health services, overall, 90.9% (95% CI, 89.8%-91.9%) employed a full- or part-time registered nurse, 29.3% (95% CI, 27.7%-31.1%) had a school-based health center, 87.2% (95% CI, 85.8%-88.4%) administered daily medications for students with chronic conditions, 77.5% (95% CI, 75.9%-79.1%) provided case management, and 65.7% (95% CI, 63.8%-67.5%) had established insurance protocols for these students (Table 3). The prevalence of having a full- or part-time registered nurse and school-based health center was noticeably lower among small schools. Schools in the Northeast reported having a full- or part-time registered nurse and an insurance protocol for students with chronic conditions substantially more often than schools in other regions (Table 3). White majority schools reported having daily medication administration and case management for students with chronic conditions more frequently than Black or Hispanic majority schools. Details describing variation according to school-level characteristics are reported in eTable 2 in the Supplement.

**Table 3.** Percentage of Public High Schools (Grades 9-12) With Specific Health Services Overall and by Select School Characteristics: School Health Profiles Principal Survey, US, 2022

Characteristic^a^	Weighted % (95% CI)				
	Full- or part-time registered nurse^b^	School-based health center^c^	Daily medication administration for students with chronic conditions^d^	Case management for students with chronic conditions^d^	Insurance protocol for students with chronic conditions^e^
Overall, nationwide	90.9 (89.8-91.9)	29.3 (27.7-31.1)	87.2 (85.8-88.4)	77.5 (75.9-79.1)	65.7 (63.8-67.5)
School enrollment^f^					
Small	75.1 (72.0-78.0)	19.9 (17.0-23.1)	84.0 (81.2-86.5)	73.4 (70.0-76.5)	62.9 (59.3-66.3)
Medium	93.9 (92.2-95.2)	29.3 (26.9-31.9)	88.7 (86.6-90.4)	79.3 (76.9-81.6)	64.9 (62.0-67.6)
Large	95.7 (93.9-97.0)	34.0 (31.0-37.1)	87.2 (84.7-89.3)	77.7 (74.7-80.4)	67.9 (64.6-71.0)
Urbanicity					
City	93.6 (90.7-95.6)	41.8 (37.5-46.3)	88.1 (84.7-90.8)	78.8 (74.9-82.2)	70.9 (66.5-74.9)
Suburban	94.1 (91.5-95.9)	30.8 (27.5-34.3)	87.4 (84.4-89.9)	77.3 (73.7-80.5)	67.4 (63.6-71.0)
Town	92.7 (90.2-94.6)	25.3 (22.1-28.9)	85.5 (82.2-88.2)	77.2 (73.4-80.5)	63.7 (59.6-67.7)
Rural	85.9 (84.0-87.6)	22.4 (20.1-24.9)	87.2 (85.1-89.1)	77.1 (74.5-79.6)	62.0 (59.0-64.9)
Region^g^					
Northeast	98.9 (97.3-99.6)^h^	33.4 (29.7-37.3)	90.1 (87.2-92.4)	82.1 (78.6-85.1)	76.0 (72.3-79.3)
Midwest	91.6 (89.7-93.1)	24.9 (22.3-27.6)	88.4 (86.3-90.2)	78.8 (76.2-81.2)	63.9 (60.9-66.9)
South	90.2 (88.2-91.8)	28.0 (25.2-30.9)	85.8 (83.1-88.0)	74.1 (70.9-77.0)	62.2 (58.9-65.4)
West	83.7 (79.9-87.0)	34.2 (29.6-39.1)	85.2 (81.4-88.4)	77.8 (73.4-81.7)	65.1 (60.0-69.8)
Neighborhood income-to-poverty ratio					
<200%	89.5 (86.3-91.9)	36.4 (32.0-41.0)	84.8 (80.8-88.1)	75.6 (71.0-79.6)	65.5 (60.8-70.0)
200% to <300%	88.9 (87.1-90.5)	26.5 (24.0-29.2)	86.5 (84.4-88.5)	76.0 (73.4-78.4)	63.6 (60.7-66.4)
≥300%	93.2 (91.5-94.6)	29.2 (26.7-31.8)	88.6 (86.5-90.3)	79.6 (77.1-81.9)	67.5 (64.6-70.2)
Racial and ethnic composition^i^					
Black majority	93.6 (88.2-96.6)^h^	36.6 (30.1-43.7)	80.7 (73.7-86.2)	71.1 (64.0-77.2)	69.3 (62.4-75.4)
Hispanic majority	90.8 (86.6-93.7)	40.2 (34.7-46.0)	82.0 (77.1-86.1)	71.6 (65.9-76.7)	64.0 (58.0-69.6)
White majority	91.0 (89.8-92.1)	24.7 (22.9-26.6)	89.4 (87.9-90.7)	79.8 (77.9-81.5)	65.0 (62.8-67.1)
No majority	92.3 (89.0-94.7)	32.3 (27.9-37.1)	87.1 (83.3-90.2)	78.7 (74.4-82.4)	68.0 (62.8-72.7)

^a^ School characteristics data were ascertained from US Department of Education National Center for Education Statistics (NCES) 2021-2022 Common Core of Data^15^ and the NCES Education Demographic and Geographic Estimates Program.^13^

^b^ Based on the survey questions: (1) “Is there a full-time registered nurse who provides health services to students at your school? (A full-time nurse means that a nurse is at the school during all school hours, 5 days per week)” and (2) “Is there a part-time registered nurse who provides health services to students at your school? (A part-time nurse means that a nurse is at the school less than 5 days a week, less than all school hours, or both).”

^c^ Based on the survey question, “Does your school have a school-based health center that offers health services to students? (School-based health centers are places on school campus where enrolled students can receive primary care, including diagnostic and treatment services. These services are usually provided by a nurse practitioner or physician’s assistant).”

^d^ Based on the survey question, “Does your school provide the following services to students?”

^e^ Based on the survey question, “Does your school have a protocol that ensures students with a chronic condition that may require daily or emergency management (e.g., asthma, diabetes, food allergies) are enrolled in private, state, or federally funded insurance programs if eligible?”

^f^ Small indicates less than 300 students; medium, 300-999 students; large, 1000 students or more.

^g^ Defined by the US Census Bureau.^14^

^h^ Indicates complement of the estimate (1 – percentage) may be unreliable (relative standard error >30%).

^i^ Race categories exclude persons of Hispanic ethnicity. Majority represents more than 50% of enrollment across grades 9 to 12. The other majority category (includes schools with American Indian or Alaska Native, Asian, Native Hawaiian or Other Pacific Islander, 2 or more races, and unspecified race majorities) was suppressed due to sample size.

### Cardiometabolic Health Education

Among all schools, 89.0% (95% CI, 87.4%-90.3%), 95.5% (95% CI, 94.3%-96.4%), and 98.1% (95% CI, 97.3%-98.7%) provided educational information to students on chronic disease prevention, nutrition, and physical activity, respectively. However, information was only provided to families in 43.5% (95% CI, 41.3%-45.7%) of schools for chronic disease prevention, 50.9% (95% CI, 48.7%-53.1%) of schools for nutrition, and 50.8% (95% CI, 48.6%-53.0%) of schools for physical activity (Table 4). Small schools were less likely to provide educational information to parents and families on each health topic; similarly, schools in the Midwest and West tended to provide educational materials to parents and families on all health topics less frequently. The prevalence of providing chronic disease prevention educational information to families was notably higher among schools in neighborhoods with an IPR of less than 300% and those with racial and ethnic student majorities other than White (Table 4). Differences across each school-level characteristic are reported in eTable 3 in the Supplement.

**Table 4.** Percentage of Public High Schools (Grades 9-12) With Efforts to Increase Student and Family Knowledge of Cardiometabolic Health Topics Overall and by Select School Characteristics: School Health Profiles Lead Health Education Teacher Survey, US, 2022

Characteristic^a^	Educational information for students, weighted % (95% CI)^b^			Educational information for parents and families, weighted % (95% CI)^c^		
	Chronic disease prevention	Nutrition	Physical activity	Chronic disease prevention	Nutrition	Physical activity
Overall, nationwide	89.0 (87.4-90.3)	95.5 (94.3-96.4)	98.1 (97.3-98.7)	43.5 (41.3-45.7)	50.9 (48.7-53.1)	50.8 (48.6-53.0)
School enrollment^d^						
Small	83.5 (79.3-87.0)	92.5 (88.8-95.0)	96.1 (93.0-97.9)^e^	37.5 (33.0-42.2)	43.5 (39.0-48.2)	44.8 (40.2-49.5)
Medium	90.3 (87.9-92.2)	96.5 (94.7-97.7)	98.4 (97.2-99.1)	46.5 (43.3-49.8)	54.3 (51.1-57.5)	53.1 (49.8-56.3)
Large	90.2 (87.6-92.3)	95.9 (93.9-97.3)	98.8 (97.5-99.4)^e^	43.4 (39.7-47.2)	51.2 (47.4-54.9)	51.5 (47.7-55.3)
Urbanicity						
City	86.4 (82.4-89.7)	93.9 (90.5-96.2)	98.0 (95.9-99.0)^e^	47.9 (42.8-52.9)	54.0 (48.9-59.0)	53.4 (48.2-58.4)
Suburban	90.8 (87.9-93.1)	97.0 (94.9-98.2)	98.6 (97.0-99.4)^e^	40.9 (36.7-45.3)	49.7 (45.4-54.0)	50.1 (45.8-54.5)
Town	93.4 (90.3-95.5)	97.0 (94.7-98.3)	99.2 (97.8-99.7)^e^	44.3 (39.5-49.2)	52.2 (47.3-57.0)	52.5 (47.7-57.3)
Rural	86.8 (83.8-89.4)	94.5 (92.0-96.3)	97.3 (95.4-98.4)	42.4 (38.8-46.0)	49.4 (45.8-53.1)	49.0 (45.4-52.6)
Region^f^						
Northeast	92.7 (89.8-94.8)	98.0 (96.2-99.0)^e^	99.5 (99.1-99.7)	44.9 (39.9-50.1)	55.9 (50.9-60.8)	55.3 (50.2-60.2)
Midwest	94.1 (92.4-95.4)	99.0 (98.4-99.4)	99.4 (98.7-99.7)^e^	38.8 (35.8-42.0)	46.0 (42.9-49.2)	46.0 (42.9-49.3)
South	85.0 (81.8-87.7)	92.4 (89.7-94.4)	96.3 (94.3-97.6)	49.6 (45.6-53.7)	55.6 (51.5-59.6)	54.4 (50.3-58.4)
West	85.5 (80.7-89.3)	93.6 (89.7-96.1)	98.4 (95.7-99.4)^e^	37.3 (32.0-42.8)	44.5 (39.0-50.0)	46.7 (41.2-52.2)
Neighborhood income-to-poverty ratio						
<200%	86.8 (82.3-90.2)	95.5 (92.1-97.5)	97.9 (95.1-99.1)^e^	54.0 (48.6-59.4)	57.3 (51.8-62.6)	56.6 (51.1-61.9)
200% to <300%	88.8 (86.2-91.1)	95.1 (93.0-96.6)	98.0 (96.5-98.9)^e^	45.5 (42.1-49.0)	52.1 (48.6-55.5)	51.7 (48.2-55.1)
≥300%	89.8 (87.5-91.8)	95.8 (93.9-97.1)	98.3 (97.1-99.0)	37.9 (34.7-41.2)	47.6 (44.3-51.0)	48.0 (44.7-51.4)
Racial and ethnic composition^g^						
Black majority	93.8 (89.5-96.4)	97.7 (95.2-98.9)^e^	98.7 (96.1-99.5)^e^	56.9 (48.7-64.6)	58.4 (50.2-66.1)	56.8 (48.6-64.6)
Hispanic majority	82.8 (77.1-87.3)	92.0 (87.6-95.0)	97.9 (94.9-99.2)^e^	53.0 (46.3-59.6)	60.7 (54.0-67.0)	61.1 (54.4-67.4)
White majority	91.0 (89.2-92.5)	96.6 (95.2-97.6)	98.3 (97.3-99.0)	38.5 (36.1-41.1)	46.7 (44.1-49.3)	47.0 (44.4-49.6)
No majority	86.7 (81.8-90.4)	93.3 (88.7-96.1)	97.0 (93.5-98.6)^e^	46.8 (40.9-52.8)	52.0 (46.1-57.9)	50.0 (44.0-56.0)

^a^ School characteristics data were ascertained from US Department of Education National Center for Education Statistics (NCES) 2021-2022 Common Core of Data^15^ and the NCES Education Demographic and Geographic Estimates Program.^13^

^b^ Based on the survey question, “During this school year, have teachers in your school tried to increase student knowledge on each of the following topics in a required course in any of grades 6 through 12?”

^c^ Based on the survey question, “During this school year, did your school provide parents and families with health information designed to increase parent and family knowledge of each of the following topics?”

^d^ Small indicates less than 300 students; medium, 300 to 999 students; large, 1000 students or more.

^e^ Indicates complement of the estimate (1 – percentage) may be unreliable (relative standard error >30%).

^f^ Defined by the US Census Bureau.^14^

^g^ Race categories exclude persons of Hispanic ethnicity. Majority represents more than 50% of enrollment across grades 9-12. The other majority category (includes schools with American Indian or Alaska Native, Asian, Native Hawaiian or Other Pacific Islander, 2 or more races, and unspecified race majorities) was suppressed due to sample size.

## Discussion

The present survey study provides a snapshot of cardiometabolic disease education and health services available to public high school students across the US. It indicates that widespread nutrition, physical activity, and chronic disease prevention education is provided for high school students but much less frequently for parents and family members. Additionally, results for several outcomes varied according to key school-level characteristics, highlighting gaps that future school-based interventions and policies may consider to help reduce the burden of cardiometabolic conditions among US adolescents.

The differences in cardiometabolic education for students and parents across multiple sociodemographic characteristics may warrant attention because these school-based education services provide valuable information and skills to school-aged youths and their families that can improve crucial health behaviors,^16,17,18^ particularly in groups and communities where cardiometabolic conditions are more prevalent among youths.^2,19^ These services are advantageous for adolescents in high school because they help prepare them for adulthood and promote the adoption of health behaviors that can reduce risks for various cardiometabolic diseases.^17,20^ The modest and varying availability of educational information for parents and families also reveals a largely untapped gap because research suggests parental and family education may be a potential leverage point to improve physical activity, nutritional habits, and lifestyle factors among school-aged youths.^18,21^ Existing frameworks, such as the Whole School, Whole Community, Whole Child^22^ and the Comprehensive School Physical Activity Program,^23^ can provide strategies to integrate nutrition and physical activity education in school settings^24,25^ and narrow gaps in access to these resources.

While the American Academy of Pediatrics urges a minimum of 1 full-time registered professional school nurse in every school,^26^ the present study observed notable disparities in the availability of a full- or part-time registered nurse in public high schools across the country. School nurses play a critical role in helping students manage chronic conditions, including more complex cardiometabolic conditions.^27,28,29^ For children and adolescents living with type 1 diabetes, school nurses are generally the key coordinator and care provider at school; they also assist with the development of critical accommodation documents (eg, individualized education programs).^27^ Research has also shown a positive association of school nurse efforts with parental perceptions on diabetes-related outcomes among youths.^30,31^ Strategies that reduce the observed differences may particularly benefit students with complex cardiometabolic conditions that require direct care and ongoing management.

### Strengths and Limitations

The present study has several strengths and novel approaches. These include providing what is, to our knowledge, the first national assessment of school-level health policies and practices across diverse sociodemographic indicators via Profiles since the discontinuation of the School Health Policies and Practices Study (SHPPS) in 2016. Historically, SHPPS provided nationally representative data on school-based policies and programs^32,33,34^ and complemented the state- and district-level surveillance measures ascertained from Profiles.^35^ However, starting in 2020, Profiles aggregated state-level data to provide national prevalence estimates,^36^ which previous research has found to be a consistent and reliable method for estimating nationwide prevalence.^37^ In addition, the current study was able to provide insights into the presence of select school health services for specific cardiometabolic conditions (ie, diabetes, hypertension, and obesity). Moreover, by linking Profiles with NCES-CCD and EDGE, the present study was able to identify more nuanced differences between US public high schools according to racial and ethnic compositions than previously reported by SPHHP literature, which tended to rely on binary characterizations based on the percentage of White students (eg, ≤50% and >50%).^32,34^ These novel details provide a more refined view of the accessibility of valuable cardiometabolic school health resources, and collectively this study provides a blueprint for future school health surveillance assessments at the national level.

This study also has limitations that should be considered. First, the outcomes reported are based on self-report and may be subject to recall and social desirability bias, although previous research has found the Profiles survey measures to be reliable for describing school policies and practices.^38^ Second, the quality of health education programs and health services available at schools was not captured. Third, response rates for the surveys varied across states; however, Profiles nonresponse analyses have suggested minimal bias from nonresponse.^11^ Fourth, the quality of the health education and health services measured in the present study was not evaluated as part of the 2 surveys. Fifth, a small number of schools were excluded from the final analytic samples, which may have slightly influenced the observed results. Sixth, while many measures described in the current study were directly related to cardiometabolic diseases, others broadly encompassed chronic diseases and may have reflected school-based resources for common noncardiometabolic conditions (eg, asthma).

## Conclusions

Using recently developed nationwide estimates from Profiles, the present study provides an updated description of the availability of beneficial cardiometabolic disease health education and health services in US public high schools according to key sociodemographic characteristics. The observed results describe previously unreported gaps in school-based resources that can help adolescents manage and decrease risks related to conditions such as diabetes, hypertension, and obesity. Continued surveillance of these types of school-based practices and services is likely to be advantageous as select cardiometabolic conditions become more common among school-aged youths.^39,40^

## Notes

Supplement 1. eTable 1. Unadjusted and adjusted prevalence ratios of public high schools tracking and providing referrals for cardiometabolic conditions by select characteristics: School Health Profiles, Principal Survey, 2022 eTable 2. Unadjusted and adjusted prevalence ratios for availability of specific health services in public high schools by select characteristics: School Health Profiles, United States, Principal Survey, 2022 eTable 3. Unadjusted and adjusted prevalence ratios for availability of educational information on cardiometabolic health topics in public high schools by select characteristics: School Health Profiles, United States, Lead Health Education Teacher Survey, 2022

Supplement 2. Data Sharing Statement
