# Supplementary material for: Cardiometabolic Disease Education and Health Services Among Public High Schools
Source: JAMA Netw Open. 2026 Sep 25;9(9):e2636060. doi: 10.1001/jamanetworkopen.2026.36060 (PMC13615464; doi:10.1001/jamanetworkopen.2026.36060)
Supplement: Supplementary file 1 [file jamanetwopen-e2636060-s001.pdf]

Supplemental Online Content

Zaganjor I, Saelee R, Sekkarie A, et al. Cardiometabolic disease education and health services among public high schools. *JAMA Netw. Open*. 2026;9(9):e2636060.
doi:10.1001/jamanetworkopen.2026.36060

eTable 1. Unadjusted and adjusted prevalence ratios of public high schools tracking and providing referrals for cardiometabolic conditions by select characteristics: School Health Profiles, Principal Survey, 2022

eTable 2. Unadjusted and adjusted prevalence ratios for availability of specific health services in public high schools by select characteristics: School Health Profiles, United States, Principal Survey, 2022

eTable 3. Unadjusted and adjusted prevalence ratios for availability of educational information on cardiometabolic health topics in public high schools by select characteristics: School Health Profiles, United States, Lead Health Education Teacher Survey, 2022

This supplemental material has been provided by the authors to give readers additional information about their work.

© 2026 Zaganjor I et al. *JAMA Network Open*.

eTable 1. Unadjusted and adjusted prevalence ratios of public high schools tracking and providing referrals for cardiometabolic conditions by select characteristics: School Health Profiles, Principal Survey, 2022[a]

Characteristic[b]	Use school records to identify and track students with current diagnosis[c]						Provide referrals for confirmed or suspected chronic conditions[d]					
	Diabetes		Hypertension		Obesity		Diabetes		Hypertension		Obesity	
	PR (95% CI)	aPR (95% CI)	PR (95% CI)	aPR (95% CI)	PR (95% CI)	aPR (95% CI)	PR (95% CI)	aPR (95% CI)	PR (95% CI)	aPR (95% CI)	PR (95% CI)	aPR (95% CI)
School enrollment[e]												
Small	0.99 (0.97–1.01)	0.99 (0.96–1.01)	0.87 (0.81–0.94)	0.91 (0.84–0.98)	0.85 (0.75–0.96)	0.94 (0.83–1.07)	0.94 (0.86–1.03)	1.01 (0.92–1.11)	0.96 (0.87–1.06)	1.04 (0.94–1.15)	0.94 (0.84–1.05)	1.08 (0.97–1.21)
Medium	Ref	Ref	Ref	Ref	Ref	Ref	Ref	Ref	Ref	Ref	Ref	Ref
Large	1.00 (0.98–1.02)	1.01 (0.99–1.03)	0.94 (0.88–1.00)[h]	0.99 (0.92–1.06)	0.92 (0.83–1.02)	0.99 (0.89–1.11)	0.98 (0.90–1.07)	1.00 (0.91–1.10)	1.03 (0.94–1.12)	1.07 (0.96–1.18)	0.99 (0.89–1.09)	0.98 (0.87–1.09)
Urbanicity												
City	1.00 (0.98–1.02)	1.00 (0.98–1.02)	1.06 (0.98–1.15)	0.97 (0.88–1.08)	1.29 (1.13–1.46)	1.06 (0.91–1.24)	1.34 (1.21–1.47)	1.18 (1.04–1.33)	1.36 (1.22–1.51)	1.14 (1.00–1.31)[h]	1.58 (1.40–1.77)	1.33 (1.15–1.55)
Suburban	1.00 (0.98–1.02)	0.99 (0.95–1.02)	1.07 (1.00–1.15)	1.00 (0.91–1.09)	1.21 (1.07–1.37)	1.00 (0.88–1.15)	1.09 (0.99–1.20)	0.99 (0.88–1.12)	1.13 (1.01–1.25)	0.99 (0.87–1.12)	1.24 (1.10–1.40)	1.10 (0.96–1.28)
Town	1.02 (1.00–1.03)[h]	1.01 (1.00–1.03)	1.07 (0.99–1.15)	1.03 (0.96–1.11)	0.98 (0.85–1.13)	0.94 (0.82–1.07)	1.17 (1.06–1.29)	1.13 (1.02–1.25)	1.18 (1.06–1.32)	1.15 (1.03–1.28)	1.24 (1.09–1.40)	1.21 (1.06–1.37)
Rural	Ref	Ref	Ref	Ref	Ref	Ref	Ref	Ref	Ref	Ref	Ref	Ref
Region[f]												
Northeast	Ref	Ref	Ref	Ref	Ref	Ref	Ref	Ref	Ref	Ref	Ref	Ref
Midwest	0.99 (0.97–1.00)	0.98 (0.97–1.00)	0.78 (0.73–0.83)	0.79 (0.74–0.85)	0.48 (0.43–0.54)	0.50 (0.45–0.57)	0.79 (0.72–0.86)	0.82 (0.75–0.90)	0.72 (0.65–0.79)	0.74 (0.67–0.82)	0.65 (0.58–0.72)	0.70 (0.63–0.78)
South	0.98 (0.96–0.99)	0.98 (0.96–1.00)[h]	0.84 (0.79–0.90)	0.81 (0.76–0.87)	0.58 (0.52–0.64)	0.54 (0.48–0.60)	0.73 (0.67–0.81)	0.72 (0.65–0.79)	0.70 (0.64–0.78)	0.67 (0.60–0.74)	0.63 (0.57–0.71)	0.62 (0.55–0.70)
West	0.97 (0.94–0.99)	0.96 (0.93–0.99)	0.73 (0.66–0.80)	0.71 (0.65–0.79)	0.49 (0.42–0.58)	0.48 (0.41–0.57)	0.84 (0.75–0.94)	0.79 (0.70–0.89)	0.80 (0.71–0.90)	0.74 (0.65–0.84)	0.74 (0.64–0.84)	0.68 (0.59–0.79)
Neighborhood IPR												
<200%	0.99 (0.97–1.01)	1.00 (0.98–1.02)	1.10 (1.01–1.19)	1.08 (0.98–1.19)	1.11 (0.97–1.27)	1.11 (0.95–1.30)	1.14 (1.04–1.26)	1.09 (0.97–1.23)	1.18 (1.06–1.31)	1.15 (1.01–1.30)	1.19 (1.05–1.34)	1.09 (0.95–1.26)
200% – <300%	1.00 (0.98–1.02)	1.00 (0.98–1.02)	1.08 (1.01–1.14)	1.11 (1.04–1.19)	1.05 (0.95–1.17)	1.20 (1.07–1.33)	0.98 (0.91–1.07)	1.02 (0.93–1.11)	1.01 (0.93–1.11)	1.07 (0.98–1.18)	1.00 (0.91–1.11)	1.07 (0.96–1.18)
≥300%	Ref	Ref	Ref	Ref	Ref	Ref	Ref	Ref	Ref	Ref	Ref	Ref
Racial and ethnic composition[g]												
Black majority	0.95 (0.91–0.99)	0.95 (0.90–1.00)	1.17 (1.06–1.28)	1.15 (1.03–1.28)	1.47 (1.27–1.71)	1.42 (1.20–1.68)	1.14 (0.99–1.31)	1.10 (0.94–1.29)	1.21 (1.05–1.40)	1.18 (1.00–1.39)[h]	1.29 (1.10–1.51)	1.21 (1.00–1.45)[h]
Hispanic majority	0.99 (0.96–1.02)	0.99 (0.97–1.02)	1.09 (1.00–1.19)[h]	1.10 (1.00–1.21)[h]	1.20 (1.04–1.40)	1.15 (0.97–1.35)	1.26 (1.13–1.40)	1.19 (1.05–1.35)	1.29 (1.15–1.45)	1.19 (1.04–1.37)	1.48 (1.31–1.68)	1.36 (1.18–1.58)
White majority	Ref	Ref	Ref	Ref	Ref	Ref	Ref	Ref	Ref	Ref	Ref	Ref
No majority	1.00 (0.97–1.02)	1.00 (0.97–1.02)	1.02 (0.94–1.11)	1.04 (0.96–1.14)	1.14 (1.00–1.31)	1.14 (0.98–1.31)	1.17 (1.06–1.30)	1.14 (1.02–1.27)	1.21 (1.09–1.35)	1.17 (1.04–1.31)	1.28 (1.13–1.45)	1.23 (1.07–1.40)

Abbreviations: aPR: adjusted prevalence ratio; CI: confidence interval; IPR: income-to-poverty ratio; PR: prevalence ratio; Ref: referent.

a. Prevalence ratios and associated 95% CIs are weighted. Adjusted models controlled for school enrollment, urbanicity, region, neighborhood income-to-poverty ratio, and racial and ethnic composition.

b. School characteristics data were ascertained from U.S. Department of Education, National Center for Education Statistics (NCES), 2021–2022 Common Core of Data (available at https://nces.ed.gov/ccd/files.asp) and the NCES Education Demographic and Geographic Estimates program (available at https://nces.ed.gov/programs/edge).

c. Based on the survey question "Does your school routinely use school records to identify and track students with a current diagnosis of the following chronic conditions? School records might include student emergency cards, medication records, health room visit information, emergency care and daily management plans, physical exam forms, or parent notes."

d. Based on the survey question "Does your school provide referrals to any organizations or health care professionals not on school property for students diagnosed with or suspected to have any of the following chronic conditions? Include referrals to school-based health centers, even if they are located on school property."

e. Enrollment in grades 9–12: small: < 300 students; medium: 300–999 students; large: ≥ 1,000 students.

f. Defined by the U.S. Census Bureau (available at https://www.census.gov/programs-surveys/economic-census/guidance-geographies/levels.html).

g. Race categories exclude persons of Hispanic ethnicity. Majority represents >50% of enrollment across grades 9–12. "Other majority" (includes schools with Asian, American Indian or Alaska Native, Native Hawaiian or other Pacific Islander, two or more races, and unspecified race majorities) category was suppressed due to sample size.

h. Rounding may cause the presented 95% CI to appear to include the null.

© 2026 Zaganjor I et al. *JAMA Network Open*.

eTable 2. Unadjusted and adjusted prevalence ratios for availability of specific health services in public high schools by select characteristics: School Health Profiles, United States, Principal Survey, 2022[a]

Characteristic[b]	Full- or part-time registered nurse[c]		School-based health center[d]		Daily medication administration for students with chronic conditions[e]		Case management for students with chronic conditions[e]		Insurance protocol for students with chronic conditions[f]	
	PR (95% CI)	aPR (95% CI)	PR (95% CI)	aPR (95% CI)	PR (95% CI)	aPR (95% CI)	PR (95% CI)	aPR (95% CI)	PR (95% CI)	aPR (95% CI)
School enrollment[g]										
Small	0.80 (0.77–0.83)	0.79 (0.74–0.83)	0.68 (0.57–0.81)	0.74 (0.61–0.89)	0.95 (0.91–0.98)	0.94 (0.89–0.98)	0.92 (0.88–0.98)	0.91 (0.86–0.97)	0.97 (0.90–1.04)	1.00 (0.92–1.08)
Medium	Ref	Ref	Ref	Ref	Ref	Ref	Ref	Ref	Ref	Ref
Large	1.02 (1.00–1.04)	1.05 (1.03–1.08)	1.16 (1.02–1.31)	1.07 (0.93–1.23)	0.98 (0.95–1.02)	0.99 (0.95–1.03)	0.98 (0.93–1.03)	1.00 (0.95–1.05)	1.05 (0.98–1.12)	1.06 (0.99–1.14)
Urbanicity										
City	1.09 (1.05–1.13)	0.98 (0.94–1.02)	1.86 (1.60–2.17)	1.41 (1.16–1.71)	1.01 (0.97–1.05)	1.04 (0.99–1.09)	1.02 (0.96–1.08)	1.03 (0.96–1.10)	1.14 (1.06–1.23)	1.09 (0.99–1.20)
Suburban	1.10 (1.06–1.13)	0.95 (0.91–1.00)	1.37 (1.18–1.60)	1.10 (0.91–1.33)	1.00 (0.96–1.04)	1.00 (0.95–1.05)	1.00 (0.95–1.06)	0.97 (0.90–1.04)	1.09 (1.01–1.17)	1.01 (0.92–1.11)
Town	1.08 (1.05–1.11)	1.01 (0.98–1.04)	1.13 (0.95–1.34)	1.01 (0.84–1.21)	0.98 (0.94–1.02)	0.96 (0.92–1.01)	1.00 (0.95–1.06)	0.97 (0.92–1.03)	1.03 (0.95–1.11)	1.02 (0.94–1.10)
Rural	Ref	Ref	Ref	Ref	Ref	Ref	Ref	Ref	Ref	Ref
Region[h]										
Northeast	Ref	Ref	Ref	Ref	Ref	Ref	Ref	Ref	Ref	Ref
Midwest	0.93 (0.91–0.95)	0.94 (0.92–0.96)	0.74 (0.64–0.87)	0.87 (0.74–1.02)	0.98 (0.95–1.02)	0.98 (0.94–1.02)	0.96 (0.91–1.01)	0.95 (0.90–1.01)	0.84 (0.79–0.90)	0.85 (0.79–0.91)
South	0.91 (0.89–0.93)	0.90 (0.87–0.92)	0.84 (0.72–0.98)	0.83 (0.70–0.99)	0.95 (0.91–0.99)	0.97 (0.93–1.01)	0.90 (0.85–0.96)	0.91 (0.85–0.97)	0.82 (0.76–0.88)	0.82 (0.76–0.88)
West	0.85 (0.81–0.88)	0.84 (0.80–0.88)	1.02 (0.86–1.22)	0.97 (0.80–1.17)	0.95 (0.90–0.99)	0.97 (0.91–1.02)	0.95 (0.89–1.01)	0.97 (0.91–1.04)	0.86 (0.78–0.94)	0.85 (0.77–0.93)
Neighborhood IPR										
<200%	0.96 (0.93–0.99)	1.01 (0.98–1.05)	1.25 (1.07–1.45)	1.17 (0.97–1.40)	0.96 (0.91–1.00)	1.00 (0.96–1.05)	0.95 (0.89–1.01)	1.02 (0.95–1.09)	0.97 (0.90–1.05)	1.01 (0.92–1.11)
200% – <300%	0.95 (0.93–0.98)	1.01 (0.98–1.03)	0.91 (0.80, 1.04)	1.01 (0.88–1.17)	0.98 (0.95–1.01)	1.00 (0.97–1.04)	0.95 (0.91–1.00)[j]	0.99 (0.94–1.04)	0.94 (0.89–1.00)	1.00 (0.93–1.06)
≥300%	Ref	Ref	Ref	Ref	Ref	Ref	Ref	Ref	Ref	Ref
Racial and ethnic composition[i]										
Black majority	1.03 (0.98–1.08)	1.02 (0.96–1.07)	1.48 (1.21–1.81)	1.22 (0.96–1.56)	0.90 (0.83–0.98)	0.87 (0.80–0.96)	0.89 (0.81–0.98)	0.88 (0.79–0.98)	1.07 (0.97–1.18)	1.05 (0.93–1.17)
Hispanic majority	1.00 (0.96–1.04)	0.99 (0.94–1.03)	1.63 (1.39–1.91)	1.26 (1.04–1.53)	0.92 (0.87–0.97)	0.90 (0.84–0.96)	0.90 (0.83–0.97)	0.86 (0.79–0.95)	0.99 (0.89–1.09)	0.93 (0.83–1.05)
White majority	Ref	Ref	Ref	Ref	Ref	Ref	Ref	Ref	Ref	Ref
No majority	1.01 (0.98–1.05)	1.00 (0.96–1.04)	1.31 (1.11–1.54)	1.06 (0.88–1.26)	0.97 (0.93–1.02)	0.96 (0.91–1.01)	0.99 (0.93–1.04)	0.97 (0.91–1.03)	1.05 (0.97–1.13)	1.01 (0.92–1.10)

Abbreviations: aPR: adjusted prevalence ratio; CI: confidence interval; IPR: income-to-poverty ratio; PR: prevalence ratio; Ref: referent.

a. Prevalence ratios and associated 95% CIs are weighted. Adjusted models controlled for school enrollment, urbanicity, region, neighborhood income-to-poverty ratio, and racial and ethnic composition.

b. School characteristics data were ascertained from U.S. Department of Education, National Center for Education Statistics (NCES), 2021–2022 Common Core of Data (available at https://nces.ed.gov/ccd/files.asp) and the NCES Education Demographic and Geographic Estimates program (available at https://nces.ed.gov/programs/edge).

c. Based on the survey questions: 1) "Is there a full-time registered nurse who provides health services to students at your school? (A full-time nurse means that a nurse is at the school during all school hours, 5 days per week.)" and 2) "Is there a part-time registered nurse who provides health services to students at your school? (A part-time nurse means that a nurse is at the school less than 5 days a week, less than all school hours, or both.)"

e. Based on the survey question "Does your school provide the following services to students?"

f. Based on the survey question "Does your school have a protocol that ensures students with a chronic condition that may require daily or emergency management (e.g., asthma, diabetes, food allergies) are enrolled in private, state, or federally funded insurance programs if eligible?"

g. Enrollment in grades 9–12: small: < 300 students; medium: 300–999 students; large: ≥ 1,000 students.

h. Defined by the U.S. Census Bureau (available at https://www.census.gov/programs-surveys/economic-census/guidance-geographies/levels.html).

i. Race categories exclude persons of Hispanic ethnicity. Majority represents >50% of enrollment across grades 9–12. "Other majority" (includes schools with Asian, American Indian or Alaska Native, Native Hawaiian or other Pacific Islander, two or more races, and unspecified race majorities) category was suppressed due to sample size.

j. Rounding may cause the presented 95% CI to appear to include the null.

© 2026 Zaganjor I et al. *JAMA Network Open*.

eTable 3. Unadjusted and adjusted prevalence ratios for availability of educational information on cardiometabolic health topics in public high schools by select characteristics: School Health Profiles, United States, Lead Health Education Teacher Survey, 2022[a]

Characteristic[b]	Educational information for students[c]						Educational information for parents and families[d]					
	Chronic disease prevention		Nutrition		Physical activity		Chronic disease prevention		Nutrition		Physical activity	
	PR (95% CI)	aPR (95% CI)	PR (95% CI)	aPR (95% CI)	PR (95% CI)	aPR (95% CI)	PR (95% CI)	aPR (95% CI)	PR (95% CI)	aPR (95% CI)	PR (95% CI)	aPR (95% CI)
School enrollment[e]												
Small	0.93 (0.88–0.97)	0.92 (0.86–0.98)	0.96 (0.92–0.99)	0.94 (0.89–0.99)	0.98 (0.95–1.00)	0.96 (0.92–1.01)	0.81 (0.70–0.93)	0.81 (0.69–0.94)	0.80 (0.71–0.91)	0.80 (0.70–0.91)	0.84 (0.75–0.95)	0.85 (0.74–0.97)
Medium	Ref	Ref	Ref	Ref	Ref	Ref	Ref	Ref	Ref	Ref	Ref	Ref
Large	1.00 (0.96–1.04)	1.05 (1.01–1.09)	0.99 (0.97–1.02)	1.01 (0.99–1.04)	1.00 (0.99–1.02)	1.01 (0.99–1.03)	0.93 (0.84–1.04)	0.99 (0.88–1.13)	0.94 (0.86–1.04)	0.98 (0.88–1.09)	0.97 (0.88–1.07)	1.00 (0.90–1.12)
Urbanicity												
City	1.00 (0.94–1.05)	0.96 (0.90–1.02)	0.99 (0.96–1.03)	0.98 (0.93–1.02)	1.01 (0.99–1.03)	0.99 (0.95–1.03)	1.13 (0.99–1.29)	0.92 (0.78–1.09)	1.09 (0.97–1.23)	0.93 (0.80–1.08)	1.09 (0.97–1.23)	0.94 (0.81–1.09)
Suburban	1.05 (1.00–1.09)[h]	0.99 (0.93–1.04)	1.03 (1.00–1.05)	1.00 (0.97–1.03)	1.01 (1.00–1.03)	0.99 (0.96–1.02)	0.97 (0.84–1.11)	0.88 (0.76–1.03)	1.00 (0.90–1.13)	0.90 (0.78–1.03)	1.02 (0.91–1.15)	0.93 (0.81–1.06)
Town	1.08 (1.03–1.12)	1.04 (1.00–1.09)[h]	1.03 (1.00–1.06)	1.01 (0.98–1.04)	1.02 (1.00–1.04)[h]	1.01 (0.99–1.03)	1.05 (0.91–1.20)	0.97 (0.85–1.12)	1.06 (0.94–1.19)	0.99 (0.88–1.11)	1.07 (0.95–1.21)	1.01 (0.90–1.14)
Rural	Ref	Ref	Ref	Ref	Ref	Ref	Ref	Ref	Ref	Ref	Ref	Ref
Region[f]												
Northeast	Ref	Ref	Ref	Ref	Ref	Ref	Ref	Ref	Ref	Ref	Ref	Ref
Midwest	1.01 (0.98–1.05)	1.00 (0.97–1.03)	1.01 (1.00–1.02)	1.01 (0.99–1.02)	1.00 (0.99–1.00)	1.00 (0.99–1.01)	0.86 (0.75–0.99)	0.90 (0.78–1.03)	0.82 (0.74–0.92)	0.87 (0.77–0.97)	0.83 (0.74–0.93)	0.87 (0.78–0.98)
South	0.92 (0.88–0.96)	0.89 (0.85–0.94)	0.94 (0.92–0.97)	0.93 (0.90–0.96)	0.97 (0.95–0.99)	0.96 (0.93–0.98)	1.11 (0.96–1.27)	1.00 (0.86–1.16)	0.99 (0.89–1.11)	0.95 (0.84–1.07)	0.98 (0.87–1.11)	0.94 (0.83–1.07)
West	0.92 (0.87–0.98)	0.94 (0.89–0.98)	0.95 (0.92–0.99)	0.96 (0.94–0.99)	0.99 (0.97–1.01)	0.99 (0.97–1.01)	0.83 (0.69–1.00)[h]	0.75 (0.62–0.91)	0.79 (0.68–0.93)	0.72 (0.62–0.85)	0.84 (0.73–0.98)	0.77 (0.66–0.91)
Neighborhood IPR												
<200%	0.97 (0.92–1.02)	1.03 (0.99–1.08)	1.00 (0.97–1.03)	1.03 (1.00–1.06)	1.00 (0.98–1.02)	1.01 (0.99–1.03)	1.43 (1.25–1.63)	1.23 (1.05–1.45)	1.20 (1.07–1.35)	1.05 (0.91–1.21)	1.18 (1.05–1.33)	1.04 (0.91–1.20)
200% – <300%	0.99 (0.95–1.03)	1.02 (0.98–1.06)	0.99 (0.97–1.02)	1.01 (0.98–1.04)	1.00 (0.98–1.01)	1.01 (0.99–1.03)	1.20 (1.07–1.35)	1.16 (1.03–1.32)	1.09 (0.99–1.20)	1.07 (0.97–1.19)	1.08 (0.98–1.19)	1.06 (0.96–1.17)
≥300%	Ref	Ref	Ref	Ref	Ref	Ref	Ref	Ref	Ref	Ref	Ref	Ref
Racial and ethnic composition[g]												
Black majority	1.03 (0.99–1.07)	1.05 (1.01–1.09)	1.01 (0.99–1.03)	1.02 (1.00–1.04)	1.00 (0.99–1.02)	1.01 (0.99–1.03)	1.47 (1.26–1.72)	1.36 (1.13–1.64)	1.25 (1.08–1.45)	1.21 (1.02–1.44)	1.21 (1.04–1.41)	1.18 (0.99–1.41)
Hispanic majority	0.91 (0.85–0.97)	0.92 (0.85–0.99)	0.95 (0.91–0.99)	0.96 (0.91–1.01)	1.00 (0.98–1.02)	0.99 (0.97–1.02)	1.37 (1.19–1.58)	1.37 (1.17–1.60)	1.30 (1.15–1.47)	1.35 (1.18–1.54)	1.30 (1.15–1.47)	1.33 (1.17–1.52)
White majority	Ref	Ref	Ref	Ref	Ref	Ref	Ref	Ref	Ref	Ref	Ref	Ref
No majority	0.95 (0.90–1.00)	0.97 (0.92–1.02)	0.97 (0.93–1.01)	0.98 (0.95–1.02)	0.99 (0.96–1.01)	0.99 (0.97–1.02)	1.22 (1.05–1.40)	1.22 (1.05–1.43)	1.11 (0.98–1.27)	1.15 (1.00–1.31)	1.06 (0.93–1.21)	1.08 (0.94–1.25)

Abbreviations: aPR: adjusted prevalence ratio; CI: confidence interval; IPR: income-to-poverty ratio; PR: prevalence ratio; Ref: referent.

a. Prevalence ratios and associated 95% CIs are weighted. Adjusted models controlled for school enrollment, urbanicity, region, neighborhood income-to-poverty ratio, and racial and ethnic composition.

b. School characteristics data were ascertained from U.S. Department of Education, National Center for Education Statistics (NCES), 2021–2022 Common Core of Data (available at https://nces.ed.gov/ccd/files.asp) and the NCES Education Demographic and Geographic Estimates program (available at https://nces.ed.gov/programs/edge).

c. Based on the survey question "During this school year, have teachers in your school tried to increase student knowledge on each of the following topics in a required course in any of grades 6 through 12?"

d. Based on the survey question "During this school year, did your school provide parents and families with health information designed to increase parent and family knowledge of each of the following topics?"

e. Enrollment in grades 9–12: small: < 300 students; medium: 300–999 students; large: ≥ 1,000 students.

f. Defined by the U.S. Census Bureau (available at https://www.census.gov/programs-surveys/economic-census/guidance-geographies/levels.html).

g. Race categories exclude persons of Hispanic ethnicity. Majority represents >50% of enrollment across grades 9–12. "Other majority" (includes schools with Asian, American Indian or Alaska Native, Native Hawaiian or other Pacific Islander, two or more races, and unspecified race majorities) category was suppressed due to sample size.

h. Rounding may cause the presented 95% CI to appear to include the null.

© 2026 Zaganjor I et al. *JAMA Network Open*.
