# Supplementary material for: Cardiometabolic Disease Education and Health Services Among Public High Schools
Source: JAMA Netw Open. 2026 Sep 25;9(9):e2636060. doi: 10.1001/jamanetworkopen.2026.36060 (PMC13615464; doi:10.1001/jamanetworkopen.2026.36060)
Supplement: Supplementary file 2 [file jamanetwopen-e2636060-s002.pdf]

Data Sharing Statement

Zaganjor. Cardiometabolic Disease Education and Health Services Among Public High Schools. *JAMA Netw Open*. Published September 25, 2026.
doi:10.1001/jamanetworkopen.2026.36060

Data
Data available: No

Additional Information
Explanation for why data not available: The School Health Profiles dataset with unique identifiers is protected by an assurance of confidentiality and is only accessible to staff within the Division of Adolescent and School Health at CDC.
